# Diophantine triples in linear recurrences of Pisot type

**DOI:** 10.1007/s40993-018-0121-2

**Published:** 2018-06-25

**Authors:** Clemens Fuchs, Christoph Hutle, Florian Luca

**Affiliations:** 1 University of Salzburg, Hellbrunner Str. 34/I, 5020 Salzburg, Austria; 2 School of Mathematics, University of the Witwatersrand, Private Bag X3, Wits, 2050 South Africa; 3 Max Planck Institute for Mathematics, Vivatsgasse 7, 53111 Bonn, Germany; 4 Department of Mathematics, Faculty of Sciences, University of Ostrava, 30 Dubna 22, 701 03 Ostrava 1, Czech Republic

**Keywords:** Diophantine triples, Linear recurrence sequences, Diophantine equations, Application of the Subspace theorem, Primary 11D72, 11B39, Secondary 11J87

## Abstract

The study of Diophantine triples taking values in linear recurrence sequences is a variant of a problem going back to Diophantus of Alexandria which has been studied quite a lot in the past. The main questions are, as usual, about existence or finiteness of Diophantine triples in such sequences. Whilst the case of binary recurrence sequences is almost completely solved, not much was known about recurrence sequences of larger order, except for very specialised generalisations of the Fibonacci sequence. Now, we will prove that any linear recurrence sequence with the Pisot property contains only finitely many Diophantine triples, whenever the order is large and a few more not very restrictive conditions are met.

## Introduction

The problem of Diophantus of Alexandria about tuples of integers $\{a_1, a_2, a_3,$ $\dots ,a_m\}$ such that the product of each distinct two of them plus 1 always results in an integer square has already quite a long history (see [8]). It is easy to see that there are infinitely many such sets with $m=2$ since $\{a,b\}=\{r-1,r+1\}$ is a Diophantine pair for every $r\ge 2$. One of the main questions was, how many such Diophantine *m*-tuples exist for a fixed $m \ge 3$. Already Euler proved that there are infinitely many Diophantine quadruples, demonstrating it with the family

$$\begin{aligned} \{a, b, a+b+2 \sqrt{ab+1}, 4(a+ \sqrt{ab+1})(b+ \sqrt{ab+1}) \sqrt{ab+1}\} \end{aligned}$$

for *a* and *b* such that $ab + 1$ is a perfect square. For $\{a,b\}=\{r-1,r+1\}$ Euler’s extension reduces to $\{a,b,c,d\}=\{r-1,r+1,4r,16r^3-4r\}$. Much later Arkin, Hoggatt and Strauss [5] proved that every Diophantine triple can be extended to a Diophantine quadruple. More precisely, let $\{a, b, c\}$ be a Diophantine triple and

$$\begin{aligned} ab + 1 = r^2, \quad ac + 1 = s^2, \quad bc + 1 = t^2, \end{aligned}$$

where *r*, *s*, *t* are positive integers. Define

$$\begin{aligned} d_+ := a + b + c + 2abc + 2rst. \end{aligned}$$

Then $\{a, b, c, d_+\}$ is a Diophantine quadruple. Dujella proved in [9], that there are no Diophantine sextuples and also that there are only finitely many Diophantine quintuples. This result is even effective, since an upper bound of the form $\log _{10}(\log _{10}(\max \{{a_i}\})) < 26$ was given on the members of such a quintuple. It is conjectured, that there are no quintuples at all and, even stronger, that if $\{a, b, c, d\}$ is a Diophantine quadruple and $d > \max \{a, b, c\}$, then $d = d_+$. The “weaker” conjecture has recently been settled by He, Togbé and Ziegler (cf. [19]), whereas the stronger conjecture still remains open.

Now it is an interesting variation of the original problem of Diophantus to consider a linear recurrence sequence instead of the sequence of squares. So we ask for bounds *m* on the size of tuples of integers $\{a_1, a_2, a_3, \dots , a_m\}$ with $a_i a_j + 1$ being members of a given linear recurrence for $1\le i<j\le m$. We shall call this set a *Diophantine m-tuple with values in the linear recurrence* (or a Diophantine *m*-tuple in the recurrences, for short). Here, the first result was due to Fuchs, Luca and Szalay, who proved in [12] that for a binary linear recurrence sequence $(u_n)_{n \ge 0}$, there are only finitely many Diophantine triples, if certain conditions are met. The Fibonacci sequence and the Lucas sequence both satisfy these conditions and all Diophantine triples with values in these sequences were computed in [22] and [23]. Further results in this direction can be found in [2, 20] and [21]. Moreover, in [1] it is shown that there are no balancing Diophantine triples; see also [3] for a related result. In [4] it is shown that there are no Diophantine triples taking values in Pellans sequence.

The first result on linear recurrence sequences of higher order than 2 came up in 2015, when the authors jointly with Irmak and Szalay proved (see [13]) that there are only finitely many Diophantine triples with values in the Tribonacci sequence $(T_n)_{n \ge 0}$ given by

$$\begin{aligned} T_0 =T_1 =0, \quad T_2 =1, \quad T_{n+3} =T_{n+2}+T_{n+1}+T_n \quad {\text {for }} n \ge 0. \end{aligned}$$

In [17] it was shown that a Tribonacci Diophantine quadruple does not exist. A related result can be found in [18]. One year later in [14], this result was generalized to *k*-generalized Fibonacci sequences: For any integer $k \ge 3$, define $(F_n^{(k)})_{n \ge 0}$ by $F_0^{(k)}=\ldots =F_{k-2}^{(k)}=0,F_{k-1}^{(k)}=1$ and

$$\begin{aligned} F_{n+k}^{(k)}=F_{n+k-1}^{(k)}+\cdots +F_n^{(k)} \quad {\text {for }} n \ge 0. \end{aligned}$$

Then for any fixed *k*, only finitely many Diophantine triples with values in $\{F_n^{(k)}; n\ge 0\}$ exist. None of these results are constructive, since the proof uses a version of the Subspace theorem. It is not clear, whether there are any Diophantine triples with values in those sequences at all.

The result in this paper deals with a significantly larger class of linear recurrence sequences:

Let $(F_n)_{n\ge 0}$ be a sequence of integers satisfying a linear recurring relation. Assume that the recurrence is of *Pisot type*, i.e., that its characteristic polynomial is the minimal polynomial (over $\mathbb {Q}$) of a Pisot number. We denote the power sum representation (Binet formula) by $F_n = f_1 \alpha _1^n + \cdots + f_k \alpha _k^n$. Assume w.l.o.g. that $\alpha =\alpha _1$ is the Pisot number; i.e., $\alpha$ is a real algebraic integer of degree *k* satisfying $\alpha >1$ and if $\alpha _2,\ldots ,\alpha _k$ denote the conjugates of $\alpha$ over $\mathbb {Q}$ then $\max \{|\alpha _2|, \dots , |\alpha _k|\} < 1$. We remark that by a result of Mignotte (cf. [24]) it immediately follows that the sequence is non-degenerate, and that the characteristic roots are all simple and irrational.

We show that there are only finitely many triples of integers $1\le a<b<c$ such that

$$\begin{aligned} 1+ab=F_x,\quad 1+ac=F_y,\quad 1+bc=F_z \end{aligned}$$

if at least one of the following conditions holds:

- Neither the leading coefficient $f_1$ nor $f_1\alpha$ is a square in $K=\mathbb {Q}(\alpha _1, \dots , \alpha _k)$.
- $k \ge 2$ and $\alpha$ is not a unit.
- $$k\ge 4$$.

The previously treated *k*-generalized Fibonacci sequences satisfy this Pisot property and neither their leading coefficient $f_1$ nor $f_1\alpha _1$ is a square. However, the new result in this paper helps us to obtain finiteness for many more linear recurrence sequences.

For example, let us consider the irreducible polynomial $X^3 - X - 1$, which has the Pisot property. Its Pisot root $\theta := 1.3247179572 \dots$ is the smallest existing Pisot number by [6]. This number is also known as the *plastic constant*. Its corresponding linear recurrence sequence $(F_n)_{n \ge 0}$, given by $F_{n+3} = F_{n+1} + F_n$, is of Pisot type. If the initial values are not $F_0 = 6, F_1 = -9, F_2 = 2$, then neither the leading coefficient nor the leading coefficient times $\theta$ are squares in the splitting field of $X^3-X-1$ over $\mathbb {Q}$. So the theorem can be applied and we obtain, that there are only finitely many Diophantine triples with values in this sequence. However it is yet not clear, what happens in the case $F_0 = 6, F_1 = -9, F_2 = 2$.

Another example for which the theorem can be applied is the polynomial

$$\begin{aligned} X^{2k+1} - \frac{X^{2k}-1}{X - 1}. \end{aligned}$$

This polynomial defines a Pisot number of degree $2k+1$ by a result of Siegel (see [25]) and its corresponding linear recurrence sequence is of Pisot type. Independently of its initial values, the result applies to all $k \ge 2$ since the degree is sufficiently large. The same applies to

$$\begin{aligned} X^{2k+1} - \frac{X^{2k+2}-1}{X^2 - 1}, \end{aligned}$$

for $$k \ge 2$$.

Furthermore, all polynomials of the form

$$\begin{aligned} X^k(X^2-X-1) + X^2 + 1 \end{aligned}$$

are known to define Pisot numbers. So, again for $k \ge 2$ the theorem applies.

We quickly discuss the main shape of the recurrences we study in this paper. Let $(F_n)_{n\ge 0}$ be a recurrence of Pisot type as described above. Let us denote $K=\mathbb {Q}(\alpha _1,\ldots ,\alpha _k)$. Since $F_n\in \mathbb {Z}$ it follows that each element of the Galois group of *K* over $\mathbb {Q}$ permutes the summands in the power sum representation of $F_n$. Moreover, each summand is a conjugate of the leading term $f_1\alpha _1^n$ over $\mathbb {Q}$ and each conjugate of it appears exactly once in the Binet formula. Therefore $F_n$ is just the trace ${\text {Tr}}_{K/\mathbb {Q}}(f_1 \alpha _1^n)$. Since $f_1$ might not be integral, we write $f_1 = f/d$ with $d \in \mathbb {Z}$ and *f* being an integral element in *K*. Thus, conversely starting with a Pisot number $\alpha$, an integer $d \in \mathbb {Z}$ and an integral element *f* in the Galois closure *K* of $\alpha$ over $\mathbb {Q}$ such that $dF_n=\text{ Tr }_{K/\mathbb {Q}}(f\alpha ^n)$ for every $n\in \mathbb {N}$, we can easily construct further examples for which our result applies.

The proof will be given in several steps: First, a more abstract theorem is going to be proved, which guarantees the existence of an algebraic equality, that needs to be satisfied, if there were infinitely many Diophantine triples. This works on utilizing the Subspace theorem (cf. [10]) and a parametrization strategy in a similar manner to that of [14]. If the leading coefficient is not a square in $\mathbb {Q}(\alpha _1, \dots , \alpha _k)$, we obtain the contradiction quite immediately from this equality. In a second step, we will use divisibility arguments and algebraic parity considerations in order to show that this equality can also not be satisfied if the order *k* is large enough. Let us now state the results.

## The results

We start with a general and more abstract statement which gives necessary conditions in case infinitely many Diophantine triples exist. It is derived by using the Subspace theorem (cf. [10]).

### Theorem 1

Let $(F_n)_{n\ge 0}$ be a sequence of integers satisfying a linear recurrence relation of Pisot type of order $k\ge 2$. Denote its power sum representation as

$$\begin{aligned} F_n = f_1 \alpha _1^n + \cdots + f_k \alpha _k^n. \end{aligned}$$

If there are infinitely many positive integers $1< a< b < c$, such that

1 $$\begin{aligned} ab+1=F_x,\qquad ac+1=F_y,\qquad bc+1=F_z \end{aligned}$$

hold for integers *x*, *y*, *z*, then one can find fixed integers $(r_1,r_2,r_3,s_1,s_2,s_3)$ with $r_1,r_2,r_3$ positive, $\gcd (r_1,r_2,r_3)=1$ such that infinitely many of the solutions (*a*, *b*, *c*, *x*, *y*, *z*) can be parametrized as

$$\begin{aligned} x=r_1 \ell +s_1,\quad y=r_2 \ell +s_2,\quad z=r_3 \ell +s_3. \end{aligned}$$

Furthermore, following the parametrization of *x*, *y*, *z* in $\ell$, there must exist a power sum $c(\ell )$ of the form

$$\begin{aligned} c(\ell ) = \alpha _1^{(-r_1+r_2+r_3)\ell + \eta } \left( e_0+\sum _{j\in J_c} e_j \prod _{i=1}^k \alpha _i^{v_{ij} \ell } \right) \end{aligned}$$

with $\eta \in \mathbb {Z} \cup (\mathbb {Z} + 1/2)$, $J_c$ an index set, $e_j$ being coefficients in $\mathbb {Q}(\alpha _1, \ldots , \alpha _k)$ and integers $v_{ij}$ with the property that $v_{ij} \ge 0$ if $i \in \{2, \dots , n\}$ and $v_{ij} < 0$ if $i = 1$, all independent of $\ell$, such that

$$\begin{aligned} (F_x - 1) c(\ell )^2 = (F_y - 1) (F_z - 1). \end{aligned}$$

Similarly there are $a(\ell )$ and $b(\ell )$ of the same shape with

$$\begin{aligned} (F_z - 1) a(\ell )^2 = (F_x - 1) (F_y - 1) \quad {\text {and}} \quad (F_y - 1) b(\ell )^2 = (F_x - 1) (F_z - 1). \end{aligned}$$

The proof is given in Sect. 4.

This theorem looks quite abstract. However, it can be applied to a huge family of linear recurrences. Firstly, it can be applied to all linear recurrences, in which the leading coefficient is not a square:

### Theorem 2

Let $(F_n)_{n \ge 0}$ be a sequence of integers satisfying a linear recurring relation $F_{n+k} = A_1 \, F_{n+k-1} + A_2 \, F_{n+k-2} + \cdots + A_k \, F_n$ of Pisot type of order $k \ge 2$, that is, the characteristic polynomial

$$\begin{aligned} X^k - A_1 \, X^{k-1} - A_2 \, X^{k-2} - \cdots - A_k = (X - \alpha _1) \, (X - \alpha _2) \, \cdots \, (X - \alpha _k) \end{aligned}$$

is an irreducible polynomial of degree *k*, has integer coefficients $A_i$, and has roots satisfying $\alpha _1 > 1$ and $\max \{ |\alpha _2|, \, \dots , \, |\alpha _k| \} < 1$. If furthermore neither $f_1$ nor $f_1 \alpha _1$ are squares in $\mathbb {Q}(\alpha _1, \dots , \alpha _k)$, then there are only finitely many Diophantine triples with values in $\{F_n; n\ge 0\}$.

The proof of this theorem is given in Sect. 5.

Another consequence of Theorem 1 applies to linear recurrences of sufficiently large order. Namely if $k \ge 4$, the existence of such a $c(\ell )$ leads to a contradiction. The same holds already for $k = 2,3$, if we assume that the Pisot element $\alpha _1$ is not a unit in the ring of integers of $\mathbb {Q}(\alpha _1,\ldots ,\alpha _k)$. Thus, we obtain the following result.

### Theorem 3

Let $(F_n)_{n \ge 0}$ be a sequence of integers satisfying a linear recurring relation $F_{n+k} = A_1 \, F_{n+k-1} + A_2 \, F_{n+k-2} + \cdots + A_k \, F_n$ of Pisot type of order $k \ge 2$, that is, the characteristic polynomial

$$\begin{aligned} X^k - A_1 \, X^{k-1} - A_2 \, X^{k-2} - \cdots - A_k = (X - \alpha _1) \, (X - \alpha _2) \, \cdots \, (X - \alpha _k) \end{aligned}$$

is an irreducible polynomial of degree *k*, has integer coefficients $A_i$, and has roots satisfying $\alpha _1 > 1$ and $\max \{ |\alpha _2|, \, \dots , \, |\alpha _k| \} < 1$. Then there are only finitely many Diophantine triples $1<a<b<c$ with

$$\begin{aligned} ab+1=F_x,\quad ac+1=F_y,\quad bc+1=F_z, \end{aligned}$$

with values in $\{F_n; n\ge 0\}$ if one of the following conditions holds:

(i) $k\ge 2$ and $\alpha _1$ is not a unit.
(ii) $$k\ge 4$$.

This theorem is proved in Sect. 6.

Before we give the proofs we first start with several useful lemmas that will be used in the sections afterwards.

## Some useful lemmas

Assume that we have infinitely many solutions $(x,y,z) \in \mathbb {N}^{3}$ to (1) with $1<a<b<c$. Obviously, we have $x< y < z$. First, one notices that not only for *z*, but for all three components, we necessarily have arbitrarily “large” solutions.

### Lemma 1

Let us assume, we have infinitely many solutions $(x,y,z) \in \mathbb {N}^{3}$ to (1). Then for each *N*, there are still infinitely many solutions $(x,y,z) \in \mathbb {N}^{3}$ with $x > N$.

### Proof

It is obvious that we must have arbitrarily large solutions for *y* and for *z*, since otherwise, *a*, *b*, *c* would all be bounded as well, which is an immediate contradiction to our assumption.

If we had infinitely many solutions (*x*, *y*, *z*) with $x < N$, then there is at least one fixed *x* which forms a solution with infinitely many pairs (*y*, *z*). Since $F_x = ab + 1$, we have a bound on these two variables as well and can use the same pigeon hole argument again to find fixed *a* and *b*, forming a Diophantine triple with infinitely many $c \in \mathbb {N}$.

Using these fixed *a*, *b*, we obtain from the other two equations in (1), that $b F_y - a F_z = b - a$ and therefore, the expressions $b f_1 \alpha _1^y$ and $a f_1 \alpha _1^z$ (having the largest growth rate) must be equal. So

$$\begin{aligned} \alpha _1^{z-y} = \frac{b}{a}, \end{aligned}$$

which is a constant. Hence, $z-y$ must be some constant $\rho > 0$ as well and we can write $z = y + \rho$ for our infinitely many solutions in *y* and *z*.

Using the power sum representations in $b F_y - a F_{y+\rho } = b - a$, we get

2 $$\begin{aligned} b \bigg (f_1 \alpha _1^y + \cdots + f_k \alpha _k^y\bigg ) - a \bigg (f_1 \alpha _1^{y+\rho } + \cdots + f_k \alpha _k^{y+\rho }\bigg ) = b-a. \end{aligned}$$

So the terms with the largest growth rate, which are $b f_1 \alpha _1^y$ and $a f_1 \alpha _1^{y+\rho }$, must be equal and this gives us $b = a \alpha _1^\rho$. Inserting this into (2) and cancelling on both sides gives us

$$\begin{aligned} \alpha _1^{\rho } \bigg (f_2 \alpha _2^y + \cdots + f_k \alpha _k^y\bigg ) - \bigg (f_2 \alpha _2^{y+\rho } + \cdots + f_k \alpha _k^{y+\rho }\bigg ) = \alpha _1^{\rho }-1. \end{aligned}$$

Now for $y \rightarrow \infty$, the left hand side converges to 0. The right hand side is a constant larger than 0. So this equality can not be true when *y* is large enough. This contradiction completes the proof. $\square$

Next, we prove the following result, which generalizes Proposition 1 in [13]. Observe that the upper bound depends now on *k*.

### Lemma 2

Let $y<z$ be sufficiently large. Then there is a constant $C_1$ such that

3 $$\begin{aligned} \gcd (F_y-1,F_z-1)< C_1 \alpha _1^{\frac{k}{k+1}z}. \end{aligned}$$

### Proof

Denote $g:=\gcd (F_y-1,F_z-1)$. Observe here and below that the numbers $F_x-1,F_y-1,F_z-1$ are positive integers. Furthermore, let us assume that *y* (and hence *z*) is large enough such that

$$\begin{aligned} \max \bigg \{\bigg |f_2\alpha _2^y + \dots + f_k \alpha _k^y\bigg |,\bigg |f_2\alpha _2^z + \dots + f_k \alpha _k^z\bigg |\bigg \} < 1/2. \end{aligned}$$

Let $\kappa$ be a constant to be determined later. If $y\le \kappa z$, then

4 $$\begin{aligned} g\le F_y-1<\vert f_1\vert \alpha _1^y\le \vert f_1\vert \alpha _1^{\kappa z}. \end{aligned}$$

Now let us assume that $y > \kappa z$. We denote $\lambda := z-y < (1 - \kappa ) z$. Note that

$$\begin{aligned} g\mid (F_z-1)-\alpha _1^{\lambda } (F_y-1)\qquad {\mathrm{in}}\qquad {\mathbb {Q}}(\alpha _1). \end{aligned}$$

Thus, we can write

$$\begin{aligned} g\pi =(F_z-1)-\alpha _1^{\lambda } (F_y-1), \end{aligned}$$

where $\pi$ is some algebraic integer in ${\mathbb {Q}}(\alpha _1)$. Note that the right-hand side above is not zero, for if it were, we would get $\alpha _1^{\lambda }=(F_z-1)/(F_y-1)\in {\mathbb {Q}}$, which is false for $\lambda >0$. We compute norms from ${\mathbb {Q}}(\alpha _1)$ to ${\mathbb {Q}}$. Observe that

$$\begin{aligned} \begin{aligned}&\left| (F_z-1)-\right. \left. \alpha _1^{\lambda }(F_y-1)\right| \\&\quad = \left| \big (f_1\alpha _1^z+\cdots + f_k \alpha _k^z - 1\big )-\alpha _1^{\lambda }\big (f_1\alpha _1^{y}+\cdots + f_k \alpha _k^y - 1\big )\right| \\&\quad = \left| \alpha _1^{\lambda }\big (1-f_2 \alpha _2^y - \cdots - f_k \alpha _k^y\big )-\big (1-f_2\alpha _2^z - \cdots - f_k \alpha _k^z\big )\right| \\&\quad \le \frac{3}{2} \alpha _1^{\lambda }-\frac{1}{2}<\frac{3}{2} \alpha _1^{\lambda }<\frac{3}{2} \alpha _1^{(1-\kappa )z}. \end{aligned} \end{aligned}$$

Further, let $\sigma _i$ be any Galois automorphism that maps $\alpha _1$ to $\alpha _i$. Then for $i\ge 2$, we have

$$\begin{aligned} \begin{aligned} \left| \sigma _i\left( (F_z-1)-\alpha _1^{\lambda } (F_y-1)\right) \right|&= \left| (F_z-1)-\alpha _i^{\lambda } (F_y-1)\right| \\&< F_z-1+F_y-1<\vert f_1\vert \alpha _1^{z}+\vert f_1\vert \alpha _1^{y}-1\\&< \vert f_1\vert \left( 1+\alpha _1^{-1}\right) \alpha _1^{z}\le C_2 \alpha _1^z, \end{aligned} \end{aligned}$$

with $C_2$ being a suitable constant (e.g. $C_2 = \vert f_1\vert \left( 1+\alpha _1^{-1}\right)$).

Altogether, we obtain

$$\begin{aligned} \begin{aligned} g^k&\le |N_{{\mathbb {Q}}(\alpha _1)/{\mathbb {Q}}}(g\pi )|\\&\le \left| N_{{\mathbb {Q}}(\alpha _1)/{\mathbb {Q}}}\big ((F_z-1)-\alpha _1^{\lambda } (F_y-1)\big )\right| \\&= \left| \prod _{i=1}^k \sigma _i\left( (F_z-1)-\alpha _1^{\lambda } (F_y-1)\right) \right| \\&< \frac{3}{2} \alpha _1^{(1-\kappa )z} (C_2 \alpha _1^{z})^{k-1}= C_3\alpha _1^{(k-\kappa )z}, \end{aligned} \end{aligned}$$

where $$C_3=3C_2^{k-1}/2$$. Hence,

5 $$\begin{aligned} g \le C_4 \alpha _1^{(1-\kappa /k)z} \end{aligned}$$

with $C_4=C_3^{1/k}$. In order to balance between (4) and (5), we choose $\kappa$ such that $\kappa =1-\kappa /k$, giving $\kappa =k/(k+1)$ and

$$\begin{aligned} g \le \max \{\vert f_1\vert , C_4\} \alpha _1^{\frac{k}{k+1}z} = C_1 \alpha _1^{\frac{k}{k+1}z}, \end{aligned}$$

where $C_1=\max \{\vert f_1\vert ,C_4\}$, which proves the lemma. $\square$

The next lemma states the irreducibility (over $\mathbb {C}$) of a certain polynomial. This lemma will be used in the proof of Theorem 3.

### Lemma 3

Assume that $k\ge 1$. For $n\ge 3$, and non-zero complex numbers $c_1,\ldots ,c_n$ the polynomial

$$\begin{aligned} c_1X_1^k+\cdots +c_nX_n^k\in {\mathbb {C}}[X_1,\ldots ,X_n] \end{aligned}$$

is irreducible.

### Proof

For $n=2$, we have the factorization $c_1X_1^k+c_2X_2^k=c_1\prod _{I=1}^k (X_1-d_iX_2)$, where $d_1,\ldots ,d_k$ are all the roots of $z^k+c_2/c_1=0$. This polynomial is square-free, that is it does not have multiple factors of degree $\ge 1$. In particular, for $n=3$,

$$\begin{aligned} c_1X_1^k+P(X_2,X_3)\in {\mathbb {C}}[X_2,X_3][X_1], \end{aligned}$$

is such that $P(X_2,X_3)=c_2X_2^k+c_3X_3^k$ is square-free. Let *p* be some irreducible factor of $P(X_2,X_3)$. Then the polynomial above is Eisenstein with respect to *p* (since $p^2$ does not divide $P(X_2,X_3)$), so the polynomial is irreducible. Now for $n\ge 4$ we apply induction on *n* noting that

$$\begin{aligned} c_1X_1^k+P(X_2,\ldots , X_n)\in {\mathbb {C}}[X_2,\ldots ,X_n][X_1], \end{aligned}$$

where $P(X_2,\ldots ,X_n)=c_2X_2^k+\cdots +c_nX_n^k$ is irreducible for $n\ge 4$ (by the induction hypothesis), so our polynomial is Eisenstein with respect to the prime $p:=P(X_2,\ldots ,X_n)$. This proves the lemma. $\square$

### Corollary 1

Assume that $k\ge 1$. If $n\ge 2$, the polynomial $c_1X_1^k+\cdots +c_nX_n^k-1$ is irreducible.

### Proof

Indeed, for if not, the homogenized polynomial

$$\begin{aligned} c_1X_1^k+\cdots +c_nX_n^k-X_{n+1}^k \end{aligned}$$

is reducible in ${\mathbb {C}}[X_1,\ldots ,X_{n+1}]$, which is impossible by Lemma 3. $\square$

Now we need to deal with the case when we have a Laurent-polynomial which looks as follows

$$\begin{aligned} P=c_1X_1^k+\cdots +c_nX_n^k-c_{n+1}/(X_1\cdots X_n)^k. \end{aligned}$$

Clearing up the powers of $X_i$ from the denominators and calculating $P-1$, it will be necessary for the proof of Theorem 3 to look at

$$\begin{aligned} (X_1\cdots X_n)^k\big (c_1X_1^k+\cdots +c_nX_n^k-1\big )-c_{n+1} \end{aligned}$$

which is a polynomial in ${\mathbb {C}}[X_1,\ldots ,X_n]$.

### Lemma 4

Assume that $k\ge 1$. Let $n\ge 3$ and $c_1,\ldots ,c_n$ be non-zero complex numbers. Then

$$\begin{aligned} (X_1\cdots X_n)^k\big (c_1X_1^k+\cdots +c_nX_n^k-1\big )-c_{n+1} \end{aligned}$$

is irreducible.

### Proof

We rewrite the polynomial as

$$\begin{aligned} X_1^{2k} \big (c_1(X_2\cdots X_n)^k\big ) + X_1^k (X_2\cdots X_n)^k\big (c_2X_2^k+\cdots +c_nX_{n}^k-1\big )-c_{n+1}=f\big (X_1^k\big ), \end{aligned}$$

where

$$\begin{aligned} f(X)=X^2\big (c_1(X_2\cdots X_n)^k\big )+X(X_2\cdots X_n)^k\big (c_2X_2^k+\cdots +c_nX_{n}^k-1\big )-c_{n+1}. \end{aligned}$$

By Capelli’s theorem, the given polynomial is irreducible if we succeed to show that:

(i) *f*(*X*) is irreducible over ${\mathbb {C}}[X_2,\ldots ,X_n]$;
(ii) If $\alpha$ is a root of *f*(*X*), then $\alpha$ is not of the form $\beta ^q$ for some element $\beta \in {\mathbb {C}}(X_2,\ldots ,X_n)(\alpha )$ and any $q\mid k$.

We consider it easier to work with the reciprocal polynomial

$$\begin{aligned} \begin{aligned} f^*(X)&= X^2f(1/X) \\&= -c_{n+1}X^2+X(X_2\cdots X_n)^k \big (c_2X_2^k+\cdots +c_nX_n^k-1\big )+c_1(X_2\cdots X_n)^k. \end{aligned} \end{aligned}$$

Additionally, since $-c_{n+1}f^*(X)=g(-c_{n+1}X)$, where

$$\begin{aligned} g(X)=X^2+X(X_2\cdots X_n)^k \big (c_2X_2^k+\cdots +c_nX_n^k-1\big )+c_1'(X_2\cdots X_n)^k, \end{aligned}$$

where $c_1'=-c_1c_{n+1}$, we can work with *g*(*X*) instead of $f^*(X)$. Note that (i) and (ii) hold for *f*(*X*) if and only if they hold for *g*(*X*). So, let us check parts (i) and (ii). Part (i) is easy. We just compute the discriminant of *g*(*X*):

$$\begin{aligned} \begin{aligned}&(X_2\cdots X_n)^{2k}\big (c_2X_2^k+\cdots +c_nX_n^k-1\big )^2-4c_1'(X_2\cdots X_n)^{k}\\&\quad = (X_2\cdots X_n)^{k} \left( (X_2\cdots X_n)^k\big (c_2X_2^k+\cdots +c_nX_n^k-1\big )^2-4c_1'\right) . \end{aligned} \end{aligned}$$

We show that the polynomial in parenthesis is square-free. Assume $p^2$ is a divisor of it for some irreducible polynomial *p* of positive degree. Putting

$$\begin{aligned} H:=c_2X_2^k+\cdots +c_nX_n^k-1 \end{aligned}$$

and taking derivatives with respect to $X_2$, we get that *p* divides

$$\begin{aligned} \begin{aligned}&\frac{\partial }{\partial X_2} \left( (X_2\cdots X_n)^k H^2-4c_1'\right) \\&\quad = kX_2^{k-1}(X_3\cdots X_n)^kH^2+2(X_2\cdots X_n)^k H \big (kc_2X_2^{k-1}\big )\\&\quad = kX_2^{k-1} (X_3\cdots X_n)^kH\big (H+2c_2X_2^k\big ). \end{aligned} \end{aligned}$$

Clearly, since *p* is irreducible, it is coprime to $X_2,\ldots ,X_n$ and *H*, so *p* must divide $H+2c_2X_2^k=(3c_2)X_2^k+c_3X_3^k+\cdots +c_n X_n^k-1$ and by Corollary 1, it must be associated to this last polynomial since this is irreducible. Since $n\ge 3$, the same argument using the partial derivative with respect to $X_3$ instead gives that *p* is associated to $c_2X_2^k+(3c_3)X_3^k+\cdots +X_n^k-1$ as well, a contradiction. This proves (i).

For part (ii), note that

$$\begin{aligned} \alpha = \frac{(X_2\cdots X_n)^kH+(X_2\cdots X_n)^{\lfloor k/2\rfloor } {\sqrt{\Delta }}}{2}, \end{aligned}$$

where

$$\begin{aligned} \Delta : = (X_2\cdots X_n)^r\big ((X_2\cdots X_n)^kH^2-4c_1'\big ), \end{aligned}$$

with $r=k-2\lfloor k/2\rfloor \in \{0,1\}$. Further, from what we proved above, $\Delta$ is square-free as a polynomial in ${\mathbb {C}}[X_2,\ldots ,X_n]$. Let $L:={\mathbb {C}}(X_2,\ldots ,X_n)$ and $\mathbf{x }=(X_2,\ldots ,X_n)$. We need to show that $\alpha$ is not of the form $\beta ^q$ for some prime $q\mid k$ and $\beta \in L(\alpha )$. Assume there is such $\beta$ and let it be

$$\begin{aligned} \beta =A(\mathbf{x })+B(\mathbf{x }){\sqrt{\Delta }},\quad {\mathrm{where}}\qquad A(\mathbf{x }),B(\mathbf{x })\in L. \end{aligned}$$

Since $\beta ^q=\alpha$, it follows that $\beta$ is integral over ${\mathbb {C}}[X_2,\ldots ,X_n][{\sqrt{\Delta }}]$, and since ${\sqrt{\Delta }}$ is integral over ${\mathbb {C}}[X_2,\ldots ,X_n]$, it follows that $\beta$ is integral over ${\mathbb {C}}[X_2,\ldots ,X_n]$. The same is true for $\gamma =A(\mathbf{x })-B(\mathbf{x }){\sqrt{\Delta }}$ since $\gamma ^q$ is the other root of *g*(*X*). Thus, $2A(\mathbf{x })=\beta +\gamma$ is integral over ${\mathbb {C}}[X_2,\ldots ,X_n]$, and since $A(\mathbf{x })\in L$, the fraction field of this last ring, it follows that $A(\mathbf{x })\in {\mathbb {C}}[X_2,\ldots ,X_n]$. Now the element $\beta \gamma =A(\mathbf{x })^2-B(\mathbf{x })^2{\Delta }$ is also integral over ${\mathbb {C}}[X_2,\ldots ,X_n]$, therefore so is $B(\mathbf{x })^2\Delta$. Thus, $B(\mathbf{x })^2\Delta$ is a polynomial and since $\Delta$ is square-free, it follows that $B(\mathbf{x })$ is itself a polynomial.

Now assume $q=2$. We then have

$$\begin{aligned} \begin{aligned} \frac{(X_2\cdots X_n)^kH+(X_2\ldots X_n)^{\lfloor k/2\rfloor } {\sqrt{\Delta }}}{2}&= (A(\mathbf{x })+B(\mathbf{x }){\sqrt{\Delta }})^2\\&= A(\mathbf{x })^2+B(\mathbf{x })^2\Delta +2A(\mathbf{x })B(\mathbf{x }){\sqrt{\Delta }}, \end{aligned} \end{aligned}$$

which gives

6 $$\begin{aligned} (X_2\cdots X_n)^kH=2A(\mathbf{x })^2+2B(\mathbf{x })^2\Delta ,\quad (X_2\cdots X_n)^{\lfloor k/2\rfloor } =4A(\mathbf{x })B(\mathbf{x }). \end{aligned}$$

The right equation above shows that both $A(\mathbf{x })$ and $B(\mathbf{x })$ are non-zero monomials of degree $\le \lfloor k/2\rfloor$ in each variable. Thus, ${\text {deg}}_{X_2}(A(\mathbf{x })^2)\le 2\lfloor k/2\rfloor \le k$ and ${\text {deg}}_{X_2}(B(\mathbf{x })^2\Delta )\ge {\text {deg}}_{X_2}(\Delta )\ge 3k>k\ge {\text {deg}}_{X_2}(A(\mathbf{x })^2)$, showing that

$$\begin{aligned} {\text {deg}}(2A(\mathbf{x })^2+2B(\mathbf{x })^2\Delta )={\text {deg}}(2B(\mathbf{x })^2\Delta )\ge 3k, \end{aligned}$$

so the left equation in (6) is impossible since the polynomial on the left-hand side has $X_2$-degree ${\text {deg}}_{X_2}((X_2\cdots X_n)^k H)=2k<3k$.

Assume next that $q\ge 3$. Taking the trace from $L({\sqrt{{\Delta }}})$ to *L* in the relation $\alpha =\beta ^q$, we get

$$\begin{aligned} (X_2\cdots X_n)^kH=(A(\mathbf{x })+B(\mathbf{x }){\sqrt{\Delta }})^q+(A(\mathbf{x })-B(\mathbf{x }){\sqrt{\Delta }})^q. \end{aligned}$$

The right-hand side factors into $(q+1)/2$ polynomials in ${\mathbb {C}}[X_2,\ldots ,X_n]$ as follows. For $k\in \{1,\ldots ,q\}$, let $\zeta _k=e^{\frac{2k\pi i}{q}}$. These are all the roots of $\zeta ^q=1$. Further, $\zeta _{q}=1$, and $\zeta _{q-k}=\zeta _k^{-1}$ for $k=1,\ldots ,(q-1)/2$. Thus,

$$\begin{aligned} \begin{aligned}&(A(\mathbf{x })+B(\mathbf{x }){\sqrt{\Delta }})^q+(A(\mathbf{x })-B(\mathbf{x }){\sqrt{\Delta }})^q\nonumber \\&\quad = \prod _{k=1}^q \left( (A(\mathbf{x })+B(\mathbf{x }){\sqrt{\Delta }})+\zeta _k(A(\mathbf{x })-B(\mathbf{x }){\sqrt{\Delta }})\right) \nonumber \\&\quad = 2A(\mathbf{x })\prod _{k=1}^{(q-1)/2} \prod _{\zeta \in \{\zeta _k,\zeta _k^{-1}\}} \left( (A(\mathbf{x })+B(\mathbf{x }){\sqrt{\Delta }})+\zeta (A(\mathbf{x })-B(\mathbf{x }){\sqrt{\Delta }})\right) \nonumber \\&\quad = 2A(\mathbf{x })\prod _{k=1}^{(q-1)/2} \left( \big (2+\zeta _k+\zeta _k^{-1}\big )A(\mathbf{x })^2+\big (2-\zeta _k-\zeta _k^{-1}\big )B(\mathbf{x })^2\Delta \right) . \end{aligned} \end{aligned}$$

If ${\text {deg}}_{X_2} (A(\mathbf{x })^2)\ne {\text {deg}}_{X_2} (B(\mathbf{x })^2\Delta )$, then each of the polynomials in the above product on the right has $X_2$-degree exactly

$$\begin{aligned} \max \bigg \{{\text {deg}}_{X_2} (A(\mathbf{x })^2),{\text {deg}}_{X_2} (B(\mathbf{x })^2\Delta )\bigg \}\ge {\text {deg}}_{X_2}(\Delta )\ge 3k, \end{aligned}$$

and such a polynomial cannot divide $(X_2\cdots X_n)^kH$, a polynomial of $X_2$-degree 2*k*. For the above deduction we used the fact that $B(\mathbf{x })\ne 0$, which is clear. Assume next that ${\text {deg}}_{X_2} (A(\mathbf{x })^2)= {\text {deg}}_{X_2} (B(\mathbf{x })^2\Delta )$ and let $a_0,b_0$ be the leading $X_2$-coefficients (as polynomials in ${\mathbb {C}}[X_3,\ldots ,X_n]$) of $A(\mathbf{x })^2$ and $B(\mathbf{x })^2\Delta$. Then the polynomial

$$\begin{aligned} \big (2+\zeta _k+\zeta _k^{-1}\big )A(\mathbf{x })^2+\big (2-\zeta _k-\zeta _k^{-1}\big )B(\mathbf{x })^2\Delta \end{aligned}$$

has $X_2$-degree ${\text {deg}}_{X_2}(B(\mathbf{x })^2\Delta )$ except if $(2+\zeta _k+\zeta _k^{-1})a_0=-(2-\zeta _k-\zeta _k^{-1})b_0$. If that happens then $a_0/b_0$ must be constant and determines uniquely the amount $\zeta _k+\zeta _k^{-1}=2\cos (2k\pi /q)$, and since $k\in \{1,\ldots ,(q-1)/2\}$, this in turn determines *k* uniquely as well. So, this shows that in this case there is at most one *k* in $\{1,\ldots ,(q-1)/2\}$ for which the polynomials from the product appearing in the right-most side of (7) can have $X_2$-degree less than ${\text {deg}}_{X_2}(\Delta )$, while all the other $(q-3)/2$ factors have degree at least ${\text {deg}}_{X_2}(\Delta )\ge 3k$ but such polynomials cannot be divisors of the polynomial $(X_2\cdots X_n)^k H$ of $X_2$-degree 2*k*. This shows that our equation is impossible for $q>3$. Thus, $q=3$ and we get

7 $$\begin{aligned} (X_2\ldots X_n)^kH=2A(\mathbf{x })(A(\mathbf{x })^2+3B(\mathbf{x })^2\Delta ). \end{aligned}$$

Recall that *H* is irreducible by Corollary 1. If $A(\mathbf{x })$ divides $(X_2\cdots X_n)^k$, it follows that ${\text {deg}}_{X_2}(A(\mathbf{x }))\le k$, so ${\text {deg}}_{X_2}(A(\mathbf{x })^2)\le 2k$. Thus, we deduce that ${\text {deg}}_{X_2}(A(\mathbf{x })^2+3B(\mathbf{x })^2\Delta )={\text {deg}}_{X_2}(3B(\mathbf{x })^2\Delta )\ge 3k$, and we get the same contradiction as before. Thus, $H\mid A(\mathbf{x })$, showing that

$$\begin{aligned} A(\mathbf{x })^2+3B(\mathbf{x })^2\Delta =aM, \end{aligned}$$

where *a* is some non-zero complex number and $M=X_2^{a_2}\cdots X_n^{a_n}$ is some monomial. We also have the relation

$$\begin{aligned} A(\mathbf{x })^2-B(\mathbf{x })^2\Delta =N_{L({\sqrt{\Delta }})/L}(\alpha )^{1/3}=c_1''(X_2\cdots X_n)^{k/3}=c_1''M_1, \end{aligned}$$

where $c_1''={\root 3 \of {c_1'}}$ (some cubic root of $c_1'$) and $M_1$ is also a monomial. Further, since

$$\begin{aligned} (X_2\cdots X_n)^{\lfloor k/2\rfloor }=\frac{\beta ^3-\gamma ^3}{\sqrt{\Delta }}=2B(\mathbf{x })(3A(\mathbf{x })^2+B(\mathbf{x })^2\Delta ), \end{aligned}$$

we see that $B(\mathbf{x })$ is a divisor of $(X_2\cdots X_n)^{\lfloor k/2\rfloor }$, so $B(\mathbf{x })=M_2$ is also a monomial. Thus, we get

$$\begin{aligned} \Delta =\frac{aM-c_1''M_1}{4M_2^2}. \end{aligned}$$

The right-hand side above is a polynomial and since $M,M_1,M_2$ are monomials, it follows that $M_2^2\mid M$ and $M_2^2\mid M_1$. Thus, $\Delta =cM_3+dM_4$ is a sum of two monomials with some non-zero coefficients. However, this is impossible since a quick look at $\Delta$ shows that as a polynomial in $X_2$ it has non-zero coefficients for $X_2^{3k+r},X_2^{2k+r},X_2^{k+r}$ and $X_2^r$, where $r=k-2\lfloor k/2\rfloor \in \{0,1\}$. This contradiction finishes (ii); hence, the proof. $\square$

## Proof of Theorem 1

The aim of this section is to prove Theorem 1.

### Proof

We first show that if there are infinitely many solutions to (1), then all of them can be parametrized by finitely many expressions as given in (14) for *c* below. The arguments in this section follow the arguments from [13] and [14].

From now on, we assume w.l.o.g. that $\alpha _1=|\alpha _1| > |\alpha _2| \ge \cdots \ge |\alpha _k|$.

We assume that there are infinitely many solutions to (1). Then, for each integer solution (*a*, *b*, *c*), we have

$$\begin{aligned} a = \sqrt{\frac{(F_x - 1)(F_y - 1)}{F_z - 1}}, \, b = \sqrt{\frac{(F_x - 1)(F_z - 1)}{F_y - 1}}, \, c = \sqrt{\frac{(F_y - 1)(F_z - 1)}{F_x - 1}}. \end{aligned}$$

Our first aim is to prove, that the growth-rates of these infinitely many *x*, *y* and *z* have to be the same, except for a multiplicative constant. Let us recall that we trivially have $x< y < z$ and that, by Lemma 1, the solutions of *x* need to diverge to infinity as well. We now want to prove that there exists a constant $C_5 > 0$ such that $C_5z < x$ for infinitely many triples (*x*, *y*, *z*).

In order to prove this, we choose *x* (and hence *y*, *z*) large enough. We denote by $g:= \gcd (F_y - 1, F_z - 1)$. Then we use Lemma 2 to obtain

$$\begin{aligned} \begin{aligned} \vert f_1\vert \alpha _1^{x}&> F_x-1 \ge \frac{F_x-1}{a}=b=\frac{F_z - 1}{c}\ge \frac{F_z-1}{g} \ge \frac{\vert f_1\vert \alpha _1^{z} - 2}{C_1 \alpha _1^{\frac{kz}{k+1}}}\\&\ge \frac{\vert f_1\vert }{C_1} \alpha _1^{\frac{z}{k+1}-1} > \vert f_1\vert \alpha _1^{\frac{z}{k+1}-C_6} \end{aligned} \end{aligned}$$

and hence

$$\begin{aligned} x > \frac{z}{k+1} - C_6 \end{aligned}$$

which implies $x > C_7 z$ for a suitable new constant $C_7$ (depending only on *k*) and *x*, *z* being sufficiently large.

Next, we do a Taylor series expansion for *c* which was given by

8 $$\begin{aligned} c = \sqrt{\frac{(F_y - 1)(F_z - 1)}{F_x - 1}}. \end{aligned}$$

Using the power sum representations of $F_x, F_y, F_z$, we get

$$\begin{aligned}\begin{aligned} c =&\sqrt{f_1} \alpha _1^{(-x+y+z)/2} \\&\times \left( 1 + (-1/f_1) \alpha _1^{-x} + (f_2/f_1) \alpha _2^x \alpha _1^{-x} + \dots + (f_k/f_1) \alpha _k^x \alpha _1^{-x}\right) ^{-1/2} \\&\times \left( 1 + (-1/f_1) \alpha _1^{-y} + (f_2/f_1) \alpha _2^y \alpha _1^{-y} + \dots + (f_k/f_1) \alpha _k^y \alpha _1^{-y} \right) ^{1/2} \\&\times \left( 1 + (-1/f_1) \alpha _1^{-z} + (f_2/f_1) \alpha _2^z \alpha _1^{-z} + \dots + (f_k/f_1) \alpha _k^z \alpha _1^{-z}\right) ^{1/2}. \end{aligned}\end{aligned}$$

We then use the binomial expansion to obtain

$$\begin{aligned} \begin{aligned}&\left( 1 \right. \left. + (-1/f_1) \alpha _1^{-x} + (f_2/f_1) \alpha _2^x \alpha _1^{-x} + \dots + (f_k/f_1) \alpha _k^x \alpha _1^{-x} \right) ^{1/2} \\&\quad = \sum _{j=0}^T \left( {\begin{array}{c}1/2\\ j\end{array}}\right) \bigg ((-1/f_1) \alpha _1^{-x} + (f_2/f_1) \alpha _2^x \alpha _1^{-x} + \dots + (f_k/f_1) \alpha _k^x \alpha _1^{-x} \bigg )^j \\&\qquad + \mathcal {O}(\alpha _1^{-(T+1) x}), \end{aligned} \end{aligned}$$

where $\mathcal {O}$ has the usual meaning, using estimates from [15] and where *T* is some index, which we will specify later. Let us write $\mathbf{x }:=(x,y,z)$. Since $x < z$ and $z < x/C_7$, the remainder term can also be written as $\mathcal {O}(\alpha _1^{-T \Vert \mathbf{x }\Vert /C_7})$, where $\Vert \mathbf{x }\Vert =\max \{x,y,z\}=z$. Doing the same for *y* and *z* likewise and multiplying those expressions gives

$$\begin{aligned} \begin{aligned} c&= \sqrt{f_1} \, \alpha _1^{(-x+y+z)/2} \cdot \left[ 1 + \dfrac{-1 + \sum _{p = 2}^k f_p \, \alpha _p^x}{f_1 \, \alpha _1^x} \right] ^{-1/2} \\&\quad \times \left[ 1 + \dfrac{-1 + \sum _{q = 2}^k f_q \, \alpha _q^y}{f_1 \, \alpha _1^y} \right] ^{1/2} \left[ 1 + \dfrac{-1 + \sum _{r = 2}^k f_r \, \alpha _r^z}{f_1 \, \alpha _1^z} \right] ^{1/2} \\&= \sqrt{f_1} \, \alpha _1^{(-x+y+z)/2} \\&\quad \times \left( \sum _{p_1=0}^{T} \sum _{q_1=0}^{T} \sum _{r_1=0}^{T} \sum _{p_0+ p_2+ \cdots + p_k = p_1} \sum _{q_0+ q_2+ \cdots + q_k = q_1} \sum _{r_0+ r_2+ \cdots + r_k = r_1} d_\mathbf{p,q,r } \, M_\mathbf{p,q,r } \right) \\&\quad + {\mathcal {O}} \left( \alpha _1^{T \Vert \mathbf{x } \Vert / C_9} \right) \end{aligned} \end{aligned}$$

in terms of

$$\begin{aligned} \begin{aligned} d_\mathbf{p,q,r }&= \dfrac{\left( - \tfrac{1}{2}\right) ! p_1! \, \tfrac{1}{2}! \, \tfrac{1}{2}!}{\left( -\tfrac{1}{2} - p_1\right) ! \, \left( \tfrac{1}{2} - q_1\right) ! \, \left( \tfrac{1}{2}-r_1\right) !} \\&\quad \times \dfrac{(-1)^{p_0+q_0+r_0}}{p_0! \, q_0! \, r_0!} \, f_1^{-p_1-q_1-q_1} \, \dfrac{f_2^{p_2+q_2+r_2}}{p_2! \, q_2! \, r_2!} \cdots \dfrac{f_k^{p_k+q_k+r_k}}{p_k! \, q_k! \, r_k!} \end{aligned} \end{aligned}$$

and

$$\begin{aligned} M_\mathbf{p,q,r } = \alpha _1^{-p_1 x - q_1 y - r_1 z} \ \alpha _2^{p_2 x + q_2 y + r_2 z} \ \cdots \ \alpha _k^{p_k x + q_k y + r_k z} \end{aligned}$$

where $\mathbf{p } = (p_0, p_1, \dots , p_k)$, $\mathbf{q } = (q_0, q_1, \dots , q_k)$, and $\mathbf{r } = (r_0, r_1, \dots , r_k)$ are vectors of non-negative integers satisfying

$$\begin{aligned} p_0+ p_2+ \cdots + p_k = p_1,\quad q_0+ q_2+ \cdots + q_k = q_1, \quad r_0+ r_2+ \cdots + r_k = r_1, \end{aligned}$$

and $\Vert \mathbf{p } \Vert , \, \Vert \mathbf{q } \Vert , \, \Vert \mathbf{r } \Vert \le T$. Since there are only finitely many such vectors, we may label the coefficients $d_\mathbf{p,q,r }$ and monomials $M_\mathbf{p,q,r }$ as $d_0, \, d_1, \, \dots , \, d_{n-1}$ and $M_0, \, M_1, \, \dots , \, M_{n-1}$, respectively, where we choose $d_0 = M_0 = 1$. In summary we have

9 $$\begin{aligned} c=\sqrt{f_1} \alpha _1^{(-x+y+z)/2} \left( 1 + \sum _{j=1}^{n-1} d_j M_j\right) + \mathcal {O}(\alpha _1^{-T \Vert \mathbf{x }\Vert /C_7}), \end{aligned}$$

where the integer *n* depends only on *T*, $d_j$ are non-zero coefficients in the field $K={\mathbb {Q}}(\alpha _1,\ldots ,\alpha _k)$, and $M_j$ is a monomial of the form

$$\begin{aligned} M_j=\prod _{i=1}^k \alpha _i^{L_{i,j}(\mathbf{x })}, \end{aligned}$$

in which $L_{i,j}(\mathbf{x })$ are linear forms in $\mathbf{x }\in {\mathbb {R}}^3$ with integer coefficients which are all non-negative if $i=2,\ldots ,k$ and negative if $i=1$. Set $J=\{1,\ldots ,n-1\}$. Note that each monomial $M_j$ is “small”, that is there exists a constant $\kappa > 0$ (which we can even choose independently of *k*), such that

10 $$\begin{aligned} |M_j| \le e^{-\kappa x}\qquad {\text {for all}}\qquad j\in J. \end{aligned}$$

This follows easily from the following fact: By the Pisot property of $F_n$, we can write $\alpha _1=|\alpha _1| > 1 + \zeta$ for a suitable $\zeta > 0$ (a conjecture of Lehmer asserts that $\zeta$ can be chosen to be an absolute constant). Using this notation and a suitable $\kappa$, we have

$$\begin{aligned}\begin{aligned} |M_j|&= |\alpha _1|^{L_{1,j}(\mathbf{x })} \cdot |\alpha _2|^{L_{2,j}(\mathbf{x })} \cdots |\alpha _k|^{L_{k,j}(\mathbf{x })} \\&\le (1 + \zeta )^{L_{1,j}(\mathbf{x })} \cdot 1 \cdots 1 \\&\le (1 + \zeta )^{-x} \\&\le e^{-\kappa x}\qquad {\text {for all}}\qquad j\in J. \end{aligned}\end{aligned}$$

Our next aim is to apply a version of the Subspace theorem given in [10] to show that there is a finite expansion of *c* involving terms as in (9); the version we are going to use can also be found in Section 3 of [16], whose notation - in particular the notion of heights - we follow.

We work with the field $K={\mathbb {Q}}(\alpha _1,\ldots ,\alpha _k)$ and let *S* be the finite set of places (which are normalized so that the Product Formula holds, cf. [10]), that are either infinite or in the set $\{ v \in M_K: |\alpha _1|_v \ne 1 \vee \cdots \vee |\alpha _k|_v \ne 1\}$. Observe that we may choose $\alpha _1,\ldots ,\alpha _k$ in $\mathbb {C}$ and therefore view *K* as a subfield of $\mathbb {C}$. We denote by $\vert \cdot \vert _\infty$ the unique place such that $\vert \beta \vert _\infty =\vert \beta \vert =\sqrt{\mathfrak {R}(\beta )^2+\mathfrak {I}(\beta )^2}$ for all $\beta \in \mathbb {C}$. According to whether $-x+y+z$ is even or odd, we set $\epsilon = 0$ or $\epsilon = 1$ respectively, such that $\alpha _1^{(-x+y+z-\epsilon )/2} \in K$. By going to a still infinite subset of the solutions, we may assume that $\epsilon$ is always either 0 or 1.

Using the fixed integer *n* (depending on *T*) from above, we now define $n+1$ linearly independent linear forms in indeterminants $(C, Y_0, \dots , Y_{n-1})$. For the place $\infty$ introduced above, we set

11 $$\begin{aligned} l_{0, \infty }(C, Y_0, \dots , Y_{n-1}) := C - \sqrt{f_1 \alpha _1^\epsilon } Y_0 - \sqrt{f_1 \alpha _1^\epsilon } \sum _{j=1}^{n-1} d_{j} Y_j, \end{aligned}$$

where $\epsilon \in \{0,1\}$ is as explained above, and

$$\begin{aligned} l_{i, \infty }(C, Y_0, \dots , Y_{n-1}) := Y_{i-1} \quad {\text {for }} i = 1, \dots , n. \end{aligned}$$

For all other places *v* in *S*, we define

$$\begin{aligned} l_{0, v} := C, \qquad l_{i, v} := Y_{i-1} \quad {\text { for }} i = 1, \dots , n. \end{aligned}$$

We will show, that there is some $\delta > 0$, such that the inequality

12 $$\begin{aligned} \prod _{v\in S}\prod _{i=0}^{n}\frac{\vert l_{i,v}(\mathbf{y })\vert _{v}}{\vert \mathbf{y }\vert _{v}} <\left( \prod _{v\in S}\vert \det (l_{0,v},\ldots ,l_{n,v})\vert _{v}\right) \cdot \mathcal {H}(\mathbf{y })^{-(n+1)-\delta }\, \end{aligned}$$

is satisfied for all vectors

$$\begin{aligned} \mathbf{y } = \bigg (c, \alpha _1^{(-x+y+z-\epsilon )/2}, \alpha _1^{(-x+y+z-\epsilon )/2} M_1, \dots , \alpha _1^{(-x+y+z-\epsilon )/2} M_{n-1}\bigg ). \end{aligned}$$

We shall use the notation $\mathbf{y }=(c,y_0,\ldots ,y_{n-1})$ below. The use of the correct $\epsilon \in \{0,1\}$ guarantees that these vectors are indeed in $K^{n+1}$.

First notice, that the determinant in (12) is given by

$$\begin{aligned} \det \left( \begin{matrix} 1 &{}\quad - \sqrt{f_1 \, \alpha _1^{\epsilon }} &{}\quad - \sqrt{f_1 \, \alpha _1^{\epsilon }} \, d_1 &{}\quad \cdots &{}\quad - \sqrt{f_1 \, \alpha _1^{\epsilon }} \, d_{n-1} \\ 0 &{}\quad 1 &{}\quad 0 &{}\quad \cdots &{}\quad 0 \\ 0 &{}\quad 0 &{}\quad 1 &{}\quad \cdots &{}\quad 0 \\ \vdots &{}\quad \vdots &{}\quad \vdots &{}\quad \ddots &{}\quad \vdots \\ 0 &{}\quad 0 &{}\quad 0 &{}\quad \cdots &{}\quad 1 \end{matrix} \right) \end{aligned}$$

if $v = \infty$ and by

$$\begin{aligned} \det \left( \begin{matrix} 1&{}\quad 0 &{}\quad \cdots &{}\quad 0 \\ 0&{}\quad 1&{}\quad \cdots &{}\quad 0 \\ \vdots &{}\quad \vdots &{}\quad \ddots &{}\quad \vdots \\ 0 &{}\quad 0 &{}\quad \cdots &{} \quad 1 \end{matrix} \right) \end{aligned}$$

if $v \ne \infty$. Thus ${\vert }\det (l_{0,v},\ldots ,l_{n,v})\vert _v=\vert 1\vert _v=1$ for all places *v*. Notice further, that

$$\begin{aligned} \mathcal {H}(\mathbf{y })= \prod _v\vert \mathbf{y }\vert _v=\prod _{v\in S}\vert \mathbf{y }\vert _v\prod _{v\notin S}\vert \mathbf{y }\vert _v\le \prod _{v\in S}\vert \mathbf{y }\vert _v \end{aligned}$$

since for all $v\notin S$ we have $\vert \mathbf{y }\vert _v=\max \{\vert c\vert _v,\vert y_0\vert _c,\ldots ,\vert y_{n-1}\vert _v\}\le 1$, which is a consequence of $c\in \mathbb {Z}$ and the remaining components $y_0,\ldots ,y_{n-1}$ of $\mathbf{y }$ being *S*-units (hence satisfy $\vert y_i\vert _v=1$ for all $v\notin S$). It follows that

$$\begin{aligned} 0\le \prod _{v\in S}\prod _{i=0}^n\frac{1}{\vert \mathbf{y }\vert _v}\le \mathcal {H}(\mathbf{y })^{-(n+1)}. \end{aligned}$$

Thus, for (12) it suffices to consider

$$\begin{aligned} \prod _{v\in S}\prod _{i=0}^{n}\vert l_{i,v}(\mathbf{y })\vert _{v} < \mathcal {H}(\mathbf{y })^{-\delta }, \end{aligned}$$

and the double product on the left-hand side can be split up into

$$\begin{aligned} \left| c - \sqrt{f_1 \alpha _1^\epsilon } y_0 - \sqrt{f_1 \alpha _1^\epsilon } \sum _{j=1}^{n-1} d_{j} y_j \right| _\infty \cdot \prod _{\begin{array}{c} v \in M_{K, \infty }, \\ v \ne \infty \end{array}} \vert c \vert _v \cdot \prod _{v \in S \backslash M_{K, \infty }} \vert c \vert _v \cdot \prod _{j=0}^{n-1} \prod _{v \in S} \vert y_j \vert _v. \end{aligned}$$

Now notice that the last double product equals 1 due to the Product Formula and that

$$\begin{aligned} \prod _{v \in S \backslash M_{K, \infty }} \vert c \vert _v \le 1, \end{aligned}$$

since $c \in \mathbb {Z}$. An upper bound on the number of infinite places in $K$ is *k*! and hence,

$$\begin{aligned}\begin{aligned} \prod _{\begin{array}{c} v \in M_{K, \infty }, \\ v \ne \infty \end{array}} \vert c \vert _v&< \left( \frac{(F_y - 1) (F_z - 1)}{F_x - 1} \right) ^{k!} \\&\le \bigg \vert f_1 \alpha _1^y + \cdots + f_k \alpha _k^y - 1\bigg \vert ^{k!} \bigg \vert f_1 \alpha _1^z + \cdots + f_k \alpha _k^z - 1 \bigg \vert ^{k!} \\&\le \bigg (\vert f_1\vert \cdot \alpha _1^{\Vert \mathbf{x } \Vert } - 1/2\bigg )^{2 \cdot k!} \end{aligned}\end{aligned}$$

for *y* large enough such that $|f_2 \alpha _2^y + \cdots + f_k \alpha _k^y| < 1/2$. And finally the first expression is just

$$\begin{aligned} \Big | \sqrt{f_1 \alpha _1^\epsilon } \alpha _1^{(-x+y+z-\epsilon )/2} \sum _{j \ge n} d_j M_j \Big |, \end{aligned}$$

which, by (9), is smaller than some expression of the form $C_{8} \alpha _1^{-T \Vert \mathbf{x }\Vert / C_7}$. Therefore, we have

$$\begin{aligned} \prod _{v\in S}\prod _{i=0}^{n}\vert l_{i,v}(\mathbf{y })\vert _{v} < C_{8} \alpha _1^{-\frac{T \Vert \mathbf{x }\Vert }{C_{9}}} \cdot \bigg (\vert f_1\vert \alpha _1^{\Vert \mathbf{x } \Vert } - 1/2\bigg )^{2 \cdot k!}. \end{aligned}$$

Now we choose *T* (and the corresponding *n*) large enough such that

$$\begin{aligned}\begin{aligned} C_{8} \alpha _1^{-\frac{T\Vert \mathbf{x }\Vert }{C_7}}< \alpha _1^{-\frac{T\Vert \mathbf{x }\Vert }{2C_7}}, \quad \bigg (\vert f_1\vert \alpha _1^{\Vert \mathbf{x }\Vert } - 1/2\bigg )^{2 \cdot k!}&< \alpha _1^{\frac{T\Vert \mathbf{x }\Vert }{4C_7}}. \end{aligned}\end{aligned}$$

Then we can write

13 $$\begin{aligned} \prod _{v\in S}\prod _{i=0}^{n}\vert l_{i,v}(\mathbf{y })\vert _{v} < \alpha _1^{\frac{-T \Vert \mathbf{x }\Vert }{4C_7}}. \end{aligned}$$

For the height of our vector $\mathbf{y }$, we have the estimate

$$\begin{aligned}\begin{aligned} \mathcal {H}(\mathbf{y })&\le C_{9} \cdot \mathcal {H}(c) \cdot \mathcal {H}\left( \alpha _1^\frac{-x+y+z-\epsilon }{2}\right) ^n \cdot \prod _{i=0}^{n-1} \mathcal {H}(M_i) \\&\le C_{9}\bigg (\vert f_1\vert \alpha _1^{\Vert \mathbf{x }\Vert } - 1/2\bigg )^{k!} \prod _{i=0}^{n-1} \alpha _1^{C_{10} \Vert \mathbf{x }\Vert } \\&\le \alpha _1^{C_{11} \Vert \mathbf{x }\Vert }, \end{aligned}\end{aligned}$$

with suitable constants $C_{9}, C_{10}, C_{11}$. For the second estimate, we used that

$$\begin{aligned} \mathcal {H}(M_j) \le \mathcal {H}(\alpha _1)^{C_{\alpha _1}(\mathbf{x })}\mathcal {H}(\alpha _2)^{C_{\alpha _2}(\mathbf{x })} \cdots \mathcal {H}(\alpha _k)^{C_{\alpha _k}(\mathbf{x })} \end{aligned}$$

and bounded it by the maximum of those expressions. Furthermore we have

$$\begin{aligned} \mathcal {H}\left( \alpha _1^\frac{-x+y+z-\epsilon }{2}\right) ^n \le \alpha _1^{n \Vert \mathbf{x }\Vert }, \end{aligned}$$

which just changes our constant $C_{11}$.

Now finally, the estimate

$$\begin{aligned} \alpha _1^{-\frac{T \Vert \mathbf{x }\Vert }{4C_7}} \le \alpha _1^{-\delta C_{11}\Vert \mathbf{x }\Vert } \end{aligned}$$

is satisfied provided that we pick $\delta$ small enough.

So all the conditions for the Subspace theorem are met. Since we assumed that there are infinitely many solutions (*x*, *y*, *z*) of (12), we now can conclude that all of them lie in finitely many proper linear subspaces. Therefore, there must be at least one proper linear subspace, which contains infinitely many solutions and we see that there exists a finite set $J_c$ and (new) coefficients $e_j$ (for $j\in J_c$) in $K$ such that we have

14 $$\begin{aligned} c=\alpha _1^{(-x+y+z-\epsilon )/2} \left( e_0+\sum _{j\in J_c} e_j M_j\right) \end{aligned}$$

with monomials $M_j$ as before.

Likewise, we can find finite expressions of this form for *a* and *b*.

Next we use the following parametrization lemma:

### Lemma 5

Suppose, we have infinitely many solutions for (1). Then there exists a line in $\mathbb {R}^3$ given by

$$\begin{aligned} x(t) = r_1 t + s_1 \quad y(t) = r_2 t + s_2 \quad z(t) = r_3 t + s_3 \end{aligned}$$

with rationals $r_1, r_2, r_3, s_1, s_2, s_3$, such that infinitely many of the solutions (*x*, *y*, *z*) are of the form (*x*(*n*), *y*(*n*), *z*(*n*)) for some integer *n*.

### Proof

Assume that (1) has infinitely many solutions. We already deduced in Section 4 that *c* can be written in the form

$$\begin{aligned} c=\alpha _1^{(-x+y+z-\epsilon )/2} \left( e_{c,0}+\sum _{j\in J_c} e_{c,j} M_{c,j}\right) \end{aligned}$$

with $J_c$ being a finite set, $e_{c,j}$ being coefficients in $K$ for $j \in J_c \cup \{0\}$ and $M_{c,j} = \prod _{i=1}^k \alpha _i^{L_{c,i,j}(\mathbf{x })}$ with $\mathbf{x }=(x,y,z)$. In the same manner, we can write

$$\begin{aligned} b=\alpha _1^{(x-y+z-\epsilon )/2} \left( e_{b,0}+\sum _{j\in J_b} e_{b,j} M_{b,j}\right) . \end{aligned}$$

Since $1 + bc = F_z = f_1 \alpha _1^z + \cdots + f_k \alpha _k^z$, we get

15 $$\begin{aligned} f_1 \alpha _1^z + \cdots + f_k \alpha _k^z - \alpha _1^{z-\varepsilon } \left( e_{b,0}+\sum _{j\in J_b} e_{b,j} M_{b,j}\right) \left( e_{c,0}+\sum _{j\in J_c} e_{c,j} M_{c,j}\right) = 1. \end{aligned}$$

We now pick $\beta _1, \dots , \beta _\ell$ as a basis for the multiplicative group generated by $\{\alpha _1, \dots , \alpha _k, -1\}$. We remark that each element in this group is an *S*-unit with the set *S* defined in Sect. 4. We express each $\alpha _1, \dots , \alpha _k$ as a product of $\beta _1, \dots , \beta _\ell$ and insert them into (15). We obtain a new equation of the form

16 $$\begin{aligned} \sum _{j \in J} e_j \beta _1^{L_{1,j}(\mathbf{x })} \cdots \beta _{\ell }^{L_{\ell ,j}(\mathbf{x })} = 0, \end{aligned}$$

where again *J* is some finite set, $e_j$ are new coefficients in $K$ and $L_{i,j}$ are linear forms in $\mathbf{x }$ with integer coefficients. Note that the sum on the left hand side is not zero, since it contains the summand $-1$. This is an *S*-unit equation.

We may assume that infinitely many of the solutions $\mathbf{x }$ are non-degenerate solutions of (16) by replacing the equation by a new equation given by a suitable vanishing subsum if necessary.

We may assume, that $(L_{1,i}, \dots , L_{\ell ,i}) \ne (L_{1,j}, \dots , L_{\ell ,j})$ for any $i \ne j$, because otherwise we could just merge these two terms.

Therefore for $i \ne j$, the theorem on non-degenerate solutions to *S*-unit equations (see [11]) yields that the set of

$$\begin{aligned}\beta _1^{L_{1,i}(\mathbf{x }) - L_{1,j}(\mathbf{x })} \cdots \beta _{\ell }^{L_{\ell ,i}(\mathbf{x }) - L_{\ell ,j}(\mathbf{x })}\end{aligned}$$

is contained in a finite set of numbers. Now since $\beta _1, \dots , \beta _{\ell }$ are multiplicatively independent, the exponents $(L_{1,i} - L_{1,j})(\mathbf{x }), \ldots , (L_{\ell ,i} - L_{\ell ,j})(\mathbf{x })$ take the same value for infinitely many $\mathbf{x }$. Since we assumed, that these linear forms are not all identically zero, this implies, that there is some non-trivial linear form *L* defined over $\mathbb {Q}$ and some $c\in \mathbb {Q}$ with $L(\mathbf{x }) = c$ for infinitely many $\mathbf{x }$. So there exist rationals $r_i, s_i, t_i$ for $i = 1, 2, 3$ such that we can parametrize

$$\begin{aligned} x = r_1 p + s_1 q + t_1, \quad y = r_2 p + s_2 q + t_2, \quad z = r_3 p + s_3 q + t_3 \end{aligned}$$

with infinitely many pairs $(p,q) \in \mathbb {Z}^2$.

We can assume, that $r_i, s_i, t_i$ are all integers. If not, we define $\Delta$ as the least common multiple of the denominators of $r_i, s_i$ ($i= 1, 2,3$) and let $p_0, q_0$ be such that for infinitely many pairs (*p*, *q*) we have $p \equiv p_0 \mod \Delta$ and $q \equiv q_0 \mod \Delta$. Then $p = p_0 + \Delta \lambda , q = q_0 + \Delta \mu$ and

$$\begin{aligned}\begin{aligned} x&= (r_1\Delta ) \lambda +(s_1\Delta ) \mu +(r_1p_0+s_1q_0+t_1)\\ y&= (r_2\Delta ) \lambda +(s_2\Delta ) \mu +(r_2p_0+s_2q_0+t_2)\\ z&= (r_3\Delta ) \lambda +(s_3\Delta ) \mu +(r_3p_0+s_3q_0+t_3). \end{aligned}\end{aligned}$$

Since $r_i \Delta$, $s_i \Delta$ and *x*, *y*, *z* are all integers, $r_i p_0 + s_i q_0 + t_i$ are integers as well. Replacing $r_i$ by $r_i \Delta$, $s_i$ by $s_i \Delta$ and $t_i$ by $r_i p_0 + s_i q_0 + t_i$, we can indeed assume, that all coefficients $r_i, s_i, t_i$ in our parametrization are integers.

Using a similar argument as in the beginning of the proof, we get that our equation is of the form

$$\begin{aligned} \sum _{j \in J} e'_j \beta _1^{L'_{1,j}(\mathbf{r })} \cdots \beta _{\ell }^{L'_{\ell ,j}(\mathbf{r })} = 0, \end{aligned}$$

where $\mathbf{r } := (\lambda , \mu )$, *J* is a finite set of indices, $e_j'$ are new non-zero coefficients in $K$ and $L'_{i,j}(\mathbf{r })$ are linear forms in $\mathbf{r }$ with integer coefficients. Again we may assume that we have $(L'_{1,i}(\mathbf{r }), \dots , L'_{\ell ,i}(\mathbf{r })) \ne (L'_{1,j}(\mathbf{r }), \dots , L'_{\ell ,j}(\mathbf{r }))$ for any $i \ne j$.

Applying the theorem of non-degenerate solutions to *S*-unit equations once more, we obtain a finite set of numbers $\Lambda$, such that for some $i \ne j$, we have

$$\begin{aligned} \beta _1^{(L'_{1,i} - L'_{1,j})(\mathbf{r })} \cdots \beta _{\ell }^{(L'_{\ell ,i} - L'_{\ell ,j})(\mathbf{r })} \in \Lambda . \end{aligned}$$

So every $\mathbf{r }$ lies on a finite collection of lines and since we had infinitely many $\mathbf{r }$, there must be some line, which contains infinitely many solutions, which proves our lemma. $\square$

We apply this lemma and define $\Delta$ as the least common multiple of the denominators of $r_1, r_2, r_3$. Infinitely many of our *n* will be in the same residue class modulo $\Delta$, which we shall call *r*. Writing $n = m \Delta + r$, we get

$$\begin{aligned} (x,y,z) = ((r_1 \Delta ) m + (r r_1 + s_1), (r_2 \Delta ) m + (r r_2 + s_2), (r_3 \Delta ) m + (r r_3 + s_3) ). \end{aligned}$$

Replacing *n* by *m*, $r_i$ by $r_i \Delta$ and $s_i$ by $r r_i + s$, we can even assume, that $r_i, s_i$ are integers. So we have

$$\begin{aligned} \frac{-x+y+z-\epsilon }{2} = \frac{(-r_1+r_2+r_3)m}{2} + \frac{-s_1+s_2+s_3 - \epsilon }{2}. \end{aligned}$$

This holds for infinitely many *m*, so we can choose a still infinite subset such that all of them are in the same residue class $\chi$ modulo 2 and we can write $m = 2 \ell + \chi$ with fixed $\chi \in \{0,1\}$. Thus, we have

$$\begin{aligned} \frac{-x+y+z-\epsilon }{2} = (-r_1 + r_2 + r_3) \ell + \eta , \end{aligned}$$

where $$\eta \in \mathbb {Z}$$ or $$\eta \in \mathbb {Z} + 1/2$$.

Using this representation, we can write (14) as

$$\begin{aligned} c(\ell ) = \alpha _1^{(-r_1+r_2+r_3)\ell + \eta } \left( e_0+\sum _{j\in J_c} e_j M_j\right) \end{aligned}$$

for infinitely many $\ell$, where

$$\begin{aligned} M_j=\prod _{i=1}^k \alpha _i^{L_{i,j}(\mathbf{x })}, \end{aligned}$$

and $$\mathbf{x } = \mathbf{x }(\ell ) = (x(2\ell + \chi ), y(2\ell + \chi ), z(2\ell + \chi ))$$.

So for infinitely many solutions (*x*, *y*, *z*), we have a parametrization in $\ell$, such that *c* is a power sum in this $\ell$ with its roots being products of $\alpha _1, \dots , \alpha _k$. This, together with (8) gives the functional identity

17 $$\begin{aligned} (F_x - 1) c^2 = (F_y - 1)(F_z - 1), \end{aligned}$$

which proves Theorem 1. $\square$

## Linear recurrences with nonsquare leading coefficient

The aim of this section is to prove Theorem 2.

### Proof

We prove this result by contradiction: Suppose we had infinitely many Diophantine triples in $\{F_n; n\ge 0\}$. Then we can apply Theorem 1 and obtain

18 $$\begin{aligned} c(\ell ) = \alpha _1^{(-r_1+r_2+r_3)\ell + \eta } \left( e_0+\sum _{j\in J_c} e_j M_j\right) \end{aligned}$$

for infinitely many $\ell$, where

$$\begin{aligned} M_j=\prod _{i=1}^k \alpha _i^{L_{i,j}(\mathbf{x })}, \end{aligned}$$

and $$\mathbf{x } = \mathbf{x }(\ell ) = (x(2\ell + \chi ), y(2\ell + \chi )), z(2\ell + \chi ))$$.

First we observe, that there are only finitely many solutions of (18) with $c(\ell ) = 0$. That can be shown by using the fact, that a simple non-degenerate linear recurrence has only finite zero-multiplicity (see [11] for an explicit bound). We will apply this statement here for the linear recurrence in $\ell$; it only remains to check, that no quotient of two distinct roots of the form

$$\begin{aligned}\alpha _1^{L_{1,i}(\mathbf{x }(\ell ))} \cdots \alpha _k^{L_{k,i}(\mathbf{x }(\ell ))}\end{aligned}$$

is a root of unity or, in other words, that

$$\begin{aligned} \bigg (\alpha _1^{m_1} \alpha _2^{m_2} \cdots \alpha _k^{m_k}\bigg )^n = 1 \end{aligned}$$

has no solutions in $n \in \mathbb {Z}/\{0\}$, $m_1 < 0$ and $m_i > 0$ for $i = 2, \dots , k$. But this follows at once from Mignotte’s result [24].

So, we have confirmed that $c(\ell ) \ne 0$ for still infinitely many solutions. We insert the finite expansion (18) in $\ell$ for *c* into (17). Furthermore, we use the Binet formula

19 $$\begin{aligned} F_x = f_1 \alpha _1^x + \cdots + f_k \alpha _k^x \end{aligned}$$

and write $F_x, F_y, F_z$ as power sums in *x*, *y* and *z* respectively. We get an equation of the form

$$\begin{aligned} \begin{aligned}&\bigg (f_1\alpha _1^x + \cdots + f_k \alpha _k^x - 1\bigg )\\&\qquad \times \alpha _1^{-x+y+z-\epsilon } \bigg (e_0^2 + 2e_0 e_1 \alpha _1^{-x} + 2e_0 e_2 \alpha _1^{-y} + 2e_0 e_3 \alpha _1^{-z} + e_1^2 \alpha _1^{-2x} + \cdots \bigg ) \\&\quad = \bigg (f_1 \alpha _1^y + \cdots + f_k \alpha _k^y - 1\bigg )\bigg (f_1 \alpha _1^z + \cdots + f_k \alpha _k^z - 1\bigg ), \end{aligned} \end{aligned}$$

Using the parametrization $(x,y,z) = (r_1 m + s_1, r_2 m + s_2, r_3 m + s_3)$ with $m = 2\ell$ or $m = 2\ell +1$, we have expansions in $\ell$ on both sides of (17). Since there must be infinitely many solutions in $\ell$, the largest terms on both sides have to grow at the same rate.

In order to find the largest terms, let us first note the following: If $e_0 = 0$ for infinitely many of our solutions, then the largest terms were

20 $$\begin{aligned} f_1 \alpha _1^x \alpha _1^{-x+y+z-\epsilon } e_1^2 \alpha _1^{-2x} = f_1 \alpha _1^y f_1 \alpha _1^z, \end{aligned}$$

or some even smaller expression on the left-hand side, if $e_1 = 0$ as well. Note that there could be more than one term in the expansion of *c* with the same growth rate, for example if *y* and *z* are just translates of *x* and therefore we have $\alpha _1^{-y} = \alpha _1^{-x-c} = C \alpha _1^{-x}$, but this would only change the coefficient $e_1$ which we do not know anyway. From (20), we get

$$\begin{aligned} e_1^2 \alpha _1^{-2x+y+z-\epsilon } = f_1 \alpha _1^{y+z}. \end{aligned}$$

Dividing by $\alpha _1^{y+z}$ on both sides, we see that the left-hand side converges to 0, when *x* grows to infinity (which it does by Lemma 1), while the right-hand side is the constant $f_1 \ne 0$. This is a contradiction.

So we must have that $e_0 \ne 0$ for infinitely many of our solutions. Then $e_0 \alpha _1^{(-x+y+z-\epsilon )/2}$ certainly is the largest term in the expansion of *c* and we have

$$\begin{aligned} f_1 \alpha _1^x \alpha _1^{-x+y+z-\epsilon } e_0^2 = f_1 \alpha _1^y f_1 \alpha _1^z. \end{aligned}$$

for the largest terms, which implies that $e_0^2 = f_1 \alpha _1^{\epsilon }$. But this is a contradiction, since we assumed that neither $f_1$ nor $f_1 \alpha _1$ is a square in $K$. So, the theorem is proved. $\square$

## Linear recurrences of large order

We now prove Theorem 3.

### Proof

We follow the same notation as in the proof of Theorem 1. Supposing that we have infinitely many Diophantine triples with values in $\{F_n; n\ge 0\}$, we get the functional identity

$$\begin{aligned} (F_x - 1) c(\ell )^2 = (F_y - 1) (F_z - 1), \end{aligned}$$

where $x=r_1\ell +s_1,y=r_2\ell +s_2,z=r_3\ell +s_3$, $r_1,r_2,r_3$ positive integers with gcd$(r_1,r_2,r_3)=1$ and $s_1,s_2,s_3$ integers.

We first handle (i) in the theorem. Therefore assume that $\alpha$ is not a unit. Then, by Mignotte’s result [24], there is no multiplicative dependence between the roots and thus (e.g. by using Lemma 2.1 in [7]), it follows that if we put $\mathbf{X }=(X_1,\ldots ,X_k)$ and

$$\begin{aligned} P_i(\mathbf{X })=\sum _{j=1}^k f_j \alpha _j^{s_i} X_j^{r_i}-1\in K[X_1,\ldots ,X_k]\quad {\text {for}}\quad i=1,2,3, \end{aligned}$$

then for each $h\in \{1,2,3\}$ putting *i*, *j* such that $\{h,i,j\}=\{1,2,3\}$, we have that

21 $$\begin{aligned} \frac{P_i(\mathbf{X })P_j(\mathbf{X })}{P_h(\mathbf{X })}=Q_h(\mathbf{X })^2, \end{aligned}$$

for some $Q_h(\mathbf{X })\in K[X_1^{\pm 1},\ldots ,X_k^{\pm 1}]$. For this we have to identify the exponential function $\ell \mapsto \alpha _1^\ell$ by $X_1$, $\ell \mapsto \alpha _2^\ell$ by $X_2$ and so forth. Actually, Theorem 1 shows that $Q_h(\mathbf{X })\in K[X_1^{\pm 1},X_2,\ldots ,X_k]$. Since the polynomial on the left-hand side of (21) has no pole at $X_1=0$ it follows that the Laurent-polynomial on the right-hand side is a polynomial in $X_1$ as well. This imposes some conditions on the degrees:

(P) *Parity:* $r_1+r_2+r_3\equiv 0\pmod 2$. This is clear from degree considerations since $2{{\text {deg}}_{X_1}}(Q_h)={{\text {deg}}_{X_1}}(P_i)+{{\text {deg}}_{X_1}} (P_j)-{{\text {deg}}_{X_1}} (P_h)=r_i+r_j-r_h$.

(T) *Triangular inequality:* $r_1+r_2> r_3$. It is clear that $r_1+r_2\ge r_3$, otherwise $P_1(\mathbf{X })P_2(\mathbf{X })/P_3(\mathbf{X })$ has negative degree as a polynomial in, say, $X_1$, so it cannot be a polynomial in $X_1$. To see that the inequality must be in fact strict, assume that equality holds. Then $Q_3(\mathbf{X })=q_3\in K[X_1]$. Hence,

$$\begin{aligned} P_1(\mathbf{X })P_2(\mathbf{X })=q_3^2 P_3(\mathbf{X }). \end{aligned}$$

In the left, we have the monomial $$X_1^{r_1} X_2^{r_2}$$ with non-zero coefficient $$f_1f_2 \alpha _1^{s_1} \alpha _2^{s_2}$$, whenever $$r_1<r_2$$. However, such monomials do not appear in the right above. Thus, we must have $$r_1=r_2$$, and since further we also have $$r_3=r_1+r_2$$ and $$\gcd (r_1,r_2,$$ $$r_3)=1$$, it follows that $$(r_1,r_2,r_3)=(1,1,2)$$. In this case, the coefficient of $$X_1X_2$$ in the left is

$$\begin{aligned} f_1f_2\bigg (\alpha _1^{s_1}\alpha _2^{s_2}+\alpha _2^{s_1}\alpha _1^{s_2}\bigg ), \end{aligned}$$

and this must be zero since $X_1X_2$ does not appear in $P_3(\mathbf{X })$. This shows that

$$\begin{aligned}(\alpha _1/\alpha _2)^{s_1-s_2}=-1,\end{aligned}$$

so $s_1=s_2$. But then $x=y$, which is not allowed.

Observe now that by the corollary to Lemma 3 (proved in Sect. 3) we know that the polynomials $P_i(\mathbf{X })$ are irreducible (as a polynomial in $\mathbb {C}[\mathbf{X }]$). We have that $r_1\le r_2\le r_3$. From (21) for $(i,j,h)=(1,2,3)$ it follows that $P_h(\mathbf{X })$ divides $P_i(\mathbf{X })$ or $P_j(\mathbf{X })$. By degree considerations, $P_h(\mathbf{X })$ divides $P_j(\mathbf{X })$ (otherwise $r_1=r_2=r_3$ and we can take them all to be 1, which is impossible by (P)). So, again by degree considerations, $P_h(\mathbf{X })$ and $P_j(\mathbf{X })$ are associated and $P_i(\mathbf{X })$ is a square which contradicts Lemma 3.

In case (ii) of the theorem, the identification with Laurent-polynomials (e.g. again via Lemma 2.1 in [7]) does not work in the above form. But when $\alpha$ is a unit, then we have the relation $\alpha _1 \cdots \alpha _k = \pm 1$, which allows applying a similar identification as we now explain. We insist again that by Mignotte’s result [24] there are no other multiplicative relations between $\alpha _1,\ldots ,\alpha _k$. In particular, any $k-1$ of these numbers (e.g. $\alpha _1,\alpha _2,\ldots ,\alpha _{k-1}$) are multiplicatively independent. Hence, we may identify $\ell \mapsto \alpha _1^\ell$ by $X_1$, $\ell \mapsto \alpha _2^\ell$ by $X_2$ and so forth, which implies that $\ell \mapsto \alpha _k^\ell$ must be identified with $\pm 1/(X_1\cdots X_{k-1})$. Theorem 1 shows that if we put

$$\begin{aligned} P_i(\mathbf{X })=\sum _{j=1}^{k-1} f_j \alpha _j^{s_i} X_j^{r_i}+\frac{f_k\alpha _k^{s_i}}{(X_1\cdots X_{k-1})^{r_i}}-1\in K\big [X_1^{\pm 1},\ldots ,X_{k-1}^{\pm 1}\big ] \end{aligned}$$

for $i=1,2,3$, then for each $h\in \{1,2,3\}$ putting *i*, *j* for the two indices such that $\{h,i,j\}=\{1,2,3\}$, we have that

22 $$\begin{aligned} \frac{P_i(\mathbf{X })P_j(\mathbf{X })}{P_h(\mathbf{X })}=Q_h(\mathbf{X })^2, \end{aligned}$$

for some $Q_h(\mathbf{X })\in K[X_1^{\pm 1},\ldots ,X_{k-1}^{\pm 1}]$. We clear on each side denominators and put $P_i'(\mathbf{X })=P_i(\mathbf{X })(X_1\cdots X_{k-1})^{r_i}\in K[X_1,\ldots ,X_{k-1}]$ for $i=1,2,3$. Then

$$\begin{aligned}((X_1\cdots X_{k-1})^{(r_i+r_j-r_h)/2}Q_h(\mathbf{X }))^2=\frac{P_i'(\mathbf{X })P_j'(\mathbf{X })}{P_h'(\mathbf{X })}.\end{aligned}$$

The left-hand side has no non-zero poles at $X_i$ while the right-hand side does not have either a zero or a pole at $X_i=0$ for $i=1,\ldots ,k-1$, therefore we deduce that $Q_h'(\mathbf{X })=(X_1\cdots X_{k-1})^{(r_i+r_j-r_h)/2}Q_h(\mathbf{X })$ is a polynomial. By Lemma 4 the polynomials $P_i'(\mathbf{X }),P_j'(\mathbf{X }),P_h'(\mathbf{X })$ are irreducible (as polynomials in $\mathbb {C}[\mathbf{X }]$). Repeating the arguments from above leads again to the sought contradiction. $\square$
